# An Essay on the Principles of Medicine

**Published:** 1831-09

**Authors:** E. S. Pearson

**Affiliations:** Surgeon-Apothecary.


### THE PRINCIPLES OF MEDICINE.

*An Essay on the Principles of Medicine.*
By E. S. Pearson, Esq. Surgeon-Apothecary.

Disease consists in an affection of either the action or the strength of the system. These qualities, although, so long as life continues, they must, to a certain degree, accompany each other, and although the same means act frequently upon both, so as equally to increase or diminish them, are nevertheless of an opposite and mutually antagonizing nature; the one having a constant tendency to the abatement of the other. This is exceedingly evident in the muscular and bony system: the forearm is remarkable for its activity, but, comparatively, it is very deficient in strength; the muscles of the lower jaw possess considerable strength, but the sphere of their action is very much contracted. This antagonism is also clearly observable in the system at large: a strong constitution is not easily excited to action of any kind; on the other hand, an active, sensible, susceptible constitution is inconsistent with any considerable degree of strength.

The application of these principles is of great importance in practice. Persons delicate in health, and liable on slight occasions to considerable excitement, may be preserved from the attack of disease by such means as augment their strength: by which same means, also, the constitution is frequently enabled to resist the influence of those poisonous exhalations which, in hot climates, are among the most fertile causes of danger to the health of such as pass through or dwell in them.

Disease may consist in an alteration either of the *degree* of the action or strength, or of the *kind* of action of the system: and first, of alterations in the degree of the vital actions. These are made up of two classes of functions: those of the vessels by which the body is nourished, and its redundances removed; and those of the nerves, by which the faculties of sense and motion are superadded to the living mass.

Excessive action of the vascular system is marked, in the first place, by an increase of heat; for the production of heat is the office chiefly of this system: it is denoted, moreover, by an increase of the colour of the body, by redness, this colour being owing to the blood which the vessels convey, and varying according to the quantity they hold; by an acceleration of the pulse, or stroke of the artery at the wrist, for the heart is the central regulating spring, if not the sole moving power of the vascular system; by an increase in the quantity of the secretions, or fluids, poured out to answer particular purposes of the animal economy; and lastly, and more particularly, by the character of the pain it is accompanied by, which is unremitting; for vascular action, unlike the nervous, is of a continued kind, and which, in a highly excited part, is produced by the slightest pressure.

The causes of this morbid affection are either direct or indirect: either such as act immediately upon the organs affected, or such as produce disease in them at a distance, by their influence upon others. Of the first kind are, exposure to the rays of a hot sun, and any excess in the use of internal stimuli, as wine, spirit, and the like.

The other class, constituted probably by the most common causes of excessive vascular action, are of a totally different nature: they depend upon a principle of the greatest importance in the human constitution: this is that of the balance of action, at least of vascular action; the consequence of which is, that we cannot considerably increase or diminish the excitement of one part of the body, without proportionably diminishing or increasing that of some other. Cold is the most common agent belonging to this class: its ordinary effect, when it produces disease, is to diminish the vascular action of the surface, and thus to excite that of the internal organs; and it will often, when applied to one extremity of the system, produce an excitement of vascular action in the opposite.

The remedies of excessive vascular action depending upon the nature of the causes, must, like them, be considered as either direct or indirect. Of the first kind, the most powerful is the abstraction of blood: there are various methods of effecting this, as by the application of cupping glasses, or of leeches, or by venesection. The two former are of importance in many topical affections, but the latter is the most efficacious; the effect of bleeding being, in a considerable degree, proportioned to the celerity with which the vital fluid is withdrawn: a fact to be accounted for, probably, by the greater shock which the living powers endure by a loss which the vessels have not time to accommodate themselves to. Where bleeding is employed, it should be continued until either the pulse becomes somewhat moderate, or some degree of faintness ensues, or the local pain (when such exists) abates. It is best to avoid taking blood, if possible, in the recumbent posture, that we may have the advantage of it should any dangerous degree of deliquium occur.

Purgatives constitute another remedy of considerable power, where the intestinal canal itself is not affected: they may be considered as exerting both a direct and indirect influence over vascular action: direct, inasmuch as they abstract from the circulation a large quantity of fluid nutriment; and indirect, inasmuch as they divert the action of the vessels from other parts of the body to the surface of the intestines.

Cold, when applied directly to an inflamed part, very rapidly abates its excitement. In the case of certain organs, (the eye, for instance, and the testicle,) it has been supposed to increase rather than diminish vascular action; but this idea is probably owing to the pain which its action upon the nervous and vascular textures of those parts occasions.

Cold, by the medium of water, is sometimes applied to the whole surface of the body, in the high vascular excitement of certain fevers, with the most marked advantage at times, at once putting an end to the disease; but when thus employed, the utmost caution is required. No remedy, indeed, is of so delicate a nature as the bath, whether it be cold or hot; none is less dependent for its efficacy upon its intrinsic powers, and more upon the manner and circumstances in which it is employed. This is owing to the extent and rapidity of its effects; so that the most exact knowledge is necessary of the degree to which they should be carried. The transitions of temperature, also, which patients who use it are liable to be exposed to, it is extremely difficult to guard against: it is necessary, too, that the application, whether of cold or heat, when made to the whole surface of the body, should be equal and continual, until their peculiar effects are produced. From all these causes, we may, in great measure, account for the comparative disuse of the very efficacious remedy of the bath; though, perhaps, something is owing to the trouble its employment gives. When, in fevers, there happens to be a natural perspiration present, and the heat is not steadily above the natural standard, no necessity exists for the practice of cold affusion, nor must we venture upon it when any sensation of chilliness is experienced by the patient. With these exceptions, cold, in such cases, may, in some way or other, be at all times applied to the body with advantage.

Abstinence from the excitement of food is a most potent means of diminishing vascular action: for this purpose it is, at any rate, necessary to refrain from animal food and all fermented liquors.

The effect of the remedies above enumerated may be explained by the supposition that they remove some stimulant or other from the constitution; but the following seem to repress excitement by acting upon what is termed the excitability of the system, that is, its disposition to be roused to vital action. These are nauseating medicines, or such as produce a tendency to sickness of the stomach; refrigerants, the chief of which are acids, more especially the vegetable, and what are more strictly termed sedatives, as lead, and the salts nitre and borax.

Of the indirect remedies of excessive vascular action, warmth is one of the most important. As cold is an indirect cause of this affection, heat is indirectly employed as the remedy of it; its effects being exactly correspondent to those which the former produce, relieving, when applied to the surface, the subjacent organs from determinations of blood, and the head when applied to the feet. The other remedies belonging to this class are denominated counter-irritants: these are such as repress excess of vascular action by the production of a certain degree of similar disease in another part of the body; they consist of rubefacient liniments, blisters, issues, and setons. These have certainly considerable power over topical affections: the two latter, however, it must be remembered, occasion considerable irritation, which may be more than commensurate with any advantage to be derived from them, at least to the expectations of the patient.

Plasters of a slightly stimulating nature are frequently of service in protecting the internal organs from the effects of cold; and persons of a delicate constitution should always wear flannel, or some such substance, next the skin, until they acquire strength.

Deficiency of vascular action is, in this part of the world, far less frequently found to constitute disease than the excess of it: it is, however, often met with, producing paleness, or even a greenish or yellowish colour of the skin, as in what is called the *green sickness;* stopping in amenorrhœa, a disease of a similar nature, the natural secretion of the uterus; causing faintness and a cessation of the circulation, as when the heart is affected; and coldness and sinking of the pulse, as in the commencement of a fever. In the two former of these instances, the cause of disease is abstinence from customary stimuli: in the two latter, it is apparently an impression made upon the excitability of the vascular system. Wine, spirits, spices, and nutritious food, are the appropriate remedies of this species of morbid affection. In amenorrhœa, electricity applied to the organ chiefly affected is frequently successful in restoring the menstrual secretion.

Violent, frequent, and painful contractions of the muscles of the limbs and trunk of the body, as well as of the muscular fibres of the viscera; unusual excitement by slight impressions upon the senses or the mind, as in hysteria; painful sensations, as in the tic douloureux; and a crowding of ideas upon the mind, as in mania, are the symptoms of excessive action of the nervous system. This is caused, in the first place, by irritations of various kinds, applied either to the extremities or the roots of the nerves. Undigested, indigestible, or corrupted food, wind, and vitiated bile, give rise to colic, or spasm of the muscular fibres of the intestines; and spiculæ of bone irritating the surface of the brain are sometimes the causes of epilepsy: but such affections as hysteria and the tic douloureux seem owing to some peculiarity in the excitability of the nervous structure; for there is nothing remarkable in the external causes which produce them. Cold is a very frequent cause of spasm.

The principal remedies of excessive nervous action are denominated antispasmodics, the chief of which is opium. The *modus operandi* of these substances we are altogether unacquainted with; we are only sensible of their beneficial operation.

Two species of remedies, viz. warmth and cordial stimulants, both of which are exceedingly hurtful in cases of morbid vascular excitement, but exceedingly efficacious in that of the nervous system, point out very clearly a remarkable opposition in the nature of these two kinds of disease. Cold, too, which abates vascular excitement, produces, as has been above noticed, nervous excitement. Thus it is evident that the two principal systems of the body, closely as they are connected with, and intimately as they influence one another, are yet governed by perfectly distinct laws.

Deficient nervous action is denoted by a partial, or by what appears to be a total, abolition of the faculties of sense and voluntary motion; the first case being that of palsy, the latter of apoplexy: both these seem to arise from the excitability of the nervous system being diminished or destroyed. They are, at any rate, generally caused by the pressure of blood effused, or of some other fluid secreted, or perhaps likewise effused from turgid blood-vessels in the centre of that system. Some kinds of palsy, however, as, for instance, that arising from lead, are apparently produced by a primary affection of the whole course of the nerves of the part affected.

Evacuations, abstinence, and such a degree of vascular stimulation as may prevent any accumulation of blood, are the means generally used for the cure of apoplexy and paralytic affections. Where these originate from any other cause than a turgescence of the blood-vessels, acrid stimulants, such as those of horseradish and mustard, should be used.

Excessive strength is a disease sometimes met with, though not often. The ancient *athletæ* are said to have frequently injured their constitutions by the efforts they made to outvie with one another in strength. This sort of affection is marked by a heaviness both of mind and of body, and a great degree of insensibility to all external impressions. It is frequently produced by too great a restraint of the natural evacuations; and the restoration of these to their proper condition, together with a certain degree of abstinence and exercise, generally effects the cure of it.

Debility is more commonly met with: it is the consequence of excesses in which the patient has indulged, and is the invariable effect of fevers. The remedies of it are well known: they are rest, nutritive and easily digestible food, and a peculiar class of medicines named tonics.

The task of the physician would be comparatively easy, if all the diseases he were called upon to treat were merely modifications of the natural actions of the system, and under the influence of agents which ordinarily affect it. Unfortunately he is frequently met by affections to which there exist no known analogies, and over which no known remedies exercise any control: these are the opprobria of medicine, and frequently the knife of the surgeon is had recourse to, as it were, to cut the knot which the physician is unable to untie. For several of these diseases, however, by great good fortune, specifics (as they are very appropriately termed) have been discovered: thus the action of mercury destroys that of the venereal disease; imbuing the system once with the vaccine poison renders it afterwards insensible to the more dangerous one of smallpox; and thus sulphur annihilates the irritation of the itch.

To treat any of these diseases by ordinary means alone, such as are mentioned above as the remedies of diseases consisting merely in alteration of the natural actions of the system, would be only trifling with them, and often exposing the patient to the greatest inconvenience or danger. Where we possess one, we must trust chiefly to the specific.

On the other hand, it would be the height of folly to neglect removing or palliating, by ordinary remedies, the morbid affections of an ordinary nature which these diseases produce; for such, in many cases, as in smallpox, constitute the chief part of the malady. When we are able to restrain these, the specific affection often in such cases quietly runs its course, and, after its allotted period, expires without serious mischief to the patient.

Disease is liable to great modifications, according as it affects various textures and organs, and in almost all cases requires a correspondent modification in its treatment. In inflammations of membranous parts, the pain is of an acute kind, and the pulse hard; in those of parenchymatous textures, the pain is dull, and the pulse soft. As the different laws which govern the organs of the body are almost as numerous as these organs themselves, and as between them and the general laws of the constitution, as well as between themselves, there exists very little apparent relation, that is to say, as almost all of them have sensibilities for peculiar stimuli, and are liable to be influenced by peculiar substances, from causes utterly unknown to us, we are obliged to trust here almost entirely to experience, and there is hence no end to the variety of medicines which the modifications of disease require. Here, indeed, opens the field of Practical Medicine, in which no one can be successful who has not devoted a considerable length of time to study and observation. To enter into the details of it would be foreign to the present purpose, which has been merely to arrange the principles of Theoretical Medicine; a task, however, of no small difficulty, from the uncertainty, and it may even be said infancy, of this department of science.

